# Ductal Adenocarcinoma of the Prostate Presenting With Bladder Invasion and Bone Metastasis: A Case Report

**DOI:** 10.7759/cureus.86000

**Published:** 2025-06-14

**Authors:** Vinesia L Riddi, Edward U Harahap, David Sitinjak, Ros Nirmawati

**Affiliations:** 1 Department of Anatomical Pathology, Dharmais Cancer Hospital–National Cancer Center, Jakarta, IDN; 2 Department of Urology, Dharmais Cancer Hospital–National Cancer Center, Jakarta, IDN

**Keywords:** bladder invasion, bone metastasis, ductal adenocarcinoma of the prostate, immunohistochemistry, prostate cancer variants

## Abstract

Ductal adenocarcinoma of the prostate (PDA) is a rare and aggressive variant of prostate cancer with a tendency for advanced local invasion and atypical metastatic spread. We report a case of a 54-year-old male presenting with urinary symptoms and significant weight loss. Imaging revealed a pelvic mass involving the prostate and bladder, with bone metastases to the pelvis and femur. Histopathological examination of transurethral resection specimens demonstrated ductal architecture. Immunohistochemistry showed α-Methylacyl-CoA racemase (AMACR) positivity, patchy cytokeratin 7 (CK7) expression, and negative prostate-specific antigen (PSA) and GATA-binding protein 3 (GATA3). Despite the unusual immunohistochemistry profile, the findings supported a diagnosis of PDA. The patient received radiotherapy and hormonal treatment, resulting in a marked PSA decline (PSA levels declined from 19.94 ng/mL to 0.34 ng/mL over eight months, representing a reduction of approximately 98.3%). This case highlights the diagnostic complexity of PDA, particularly when immunohistochemical findings deviate from classical patterns, and underscores the need for an integrated diagnostic approach in managing aggressive prostate cancer variants.

## Introduction

Ductal adenocarcinoma of the prostate (PDA) is an uncommon but notably aggressive histological subtype of prostate cancer, representing the second most frequent variant following the conventional acinar adenocarcinoma. Pure forms of PDA account for approximately 0.17% of all prostate cancer cases based on meta-analytical data. However, its prevalence increases to 0.4%-5% when cases with mixed ductal-acinar components are included, with higher detection rates observed in prostatectomy specimens compared to needle biopsies [1-4]. 

PDA is distinguished by unique clinical and pathological characteristics, most notably its tendency to present at more advanced local stages (T3/T4) and with distant metastases at initial diagnosis [1]. These findings are often paradoxical, as patients with PDA may exhibit disproportionately low prostate-specific antigen (PSA) levels relative to tumor burden when compared to those with acinar adenocarcinoma. This phenomenon may be attributed to the histological origin of PDA, which arises from the prostatic ducts and periurethral region rather than the secretory acini. These ductal tumor cells typically produce less PSA compared to acinar adenocarcinoma cells, which originate from PSA-secreting acinar glands. Furthermore, the loss of differentiation in more aggressive PDA may reduce PSA expression at the cellular level, contributing to relatively lower serum PSA despite significant tumor volume [1,2,5]. 

The biological aggressiveness of PDA is further demonstrated by a significantly increased risk, estimated at 4.6 times greater, of metastatic progression compared to acinar subtypes [1,5,6]. Moreover, PDA exhibits a proclivity for metastasizing to less common sites, including bone, rectum, and visceral organs. Bladder invasion, as demonstrated in this case, reflects its pattern of local aggressiveness and frequently necessitates comprehensive, multimodal treatment approaches. Although skeletal metastases are generally less prevalent in PDA than in acinar adenocarcinoma, their presence in advanced disease is associated with unfavorable prognostic outcomes [1,6].

This report describes a rare case of PDA with concurrent bladder invasion and femoral metastasis, highlighting the tumor’s aggressive clinical course and contributing to the limited body of literature on its atypical metastatic presentation.

## Case presentation

A 54-year-old male was referred to our institution with a suspected tumor involving both the prostate and bladder. Three months prior to admission, the patient experienced urinary difficulties accompanied by groin pain. His body weight had declined significantly, from a usual weight of approximately 80 kg to 57 kg at the time of referral. He had previously undergone a transurethral resection (TUR) procedure involving the prostate and bladder neck at the referring hospital. Histopathological examination of the TUR specimen at the referring hospital confirmed prostatic adenocarcinoma with ductal features. A histopathological slide review was also conducted at our center to validate the diagnosis. 

**Figure 1:**
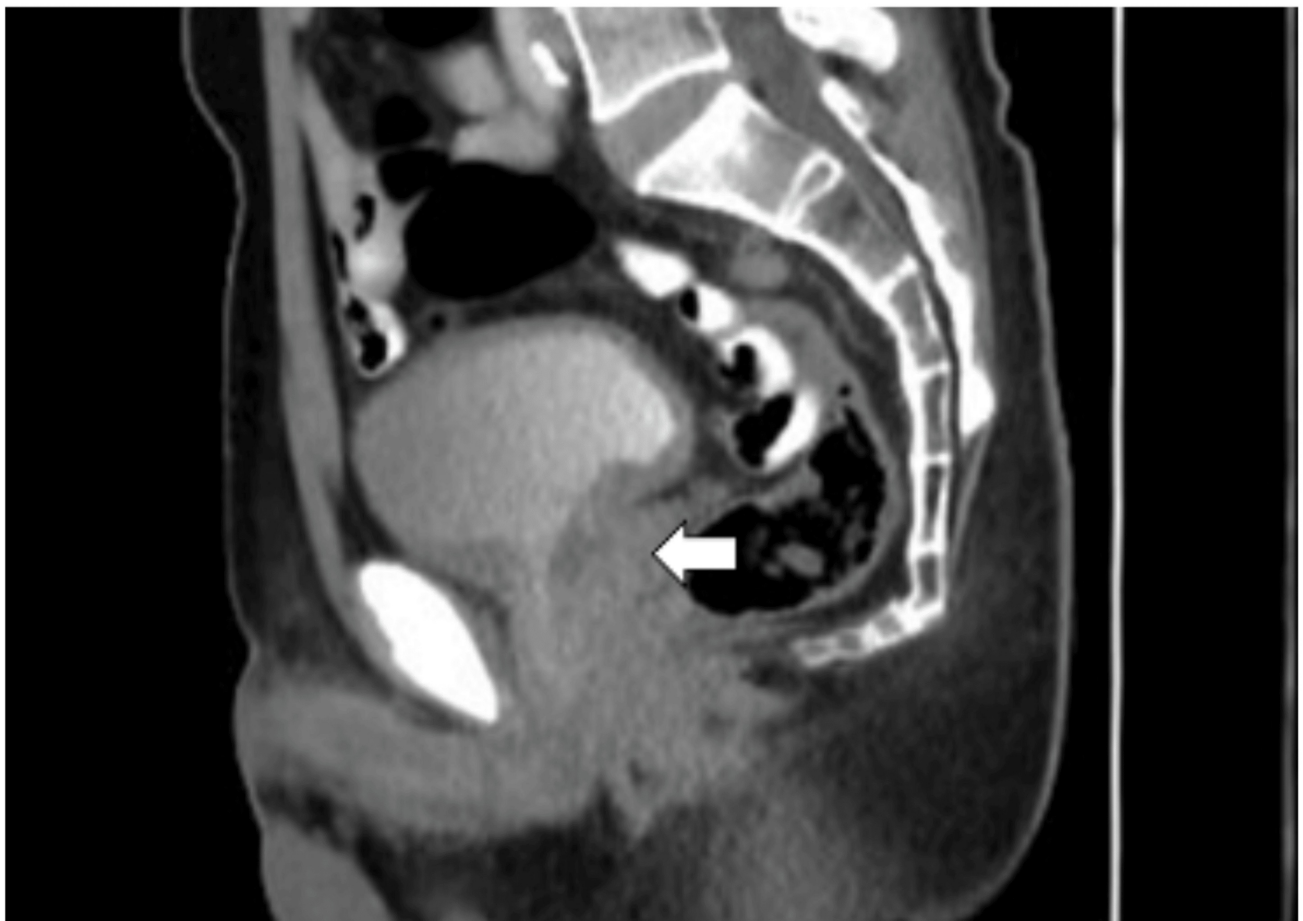
Abdominopelvic multislice computed tomography scan showing infiltrative mass (axial view). The white arrow demonstrates a solid mass involving the posteroinferior bladder wall, perivesical fat, prostate gland, and seminal vesicles.

At our institution, a baseline serum prostate-specific antigen (PSA) level measured on January 23, 2024, was elevated at 19.94 ng/mL (reference range: <4.00 ng/mL). In February 2024, an abdominopelvic multislice computed tomography (MSCT) scan performed at our institution revealed a mass measuring 3.8 x 3.4 x 4.4 cm involving the posteroinferior bladder wall, perivesical fat, prostate gland, and seminal vesicles. This mass had increased in size compared to preoperative (pre-TUR) measurements from the referring hospital of 3 x 2 x 3.5 cm. The imaging features of the lesion (post-TUR procedure) on MSCT are presented in Figure 1.

**Figure 2:**
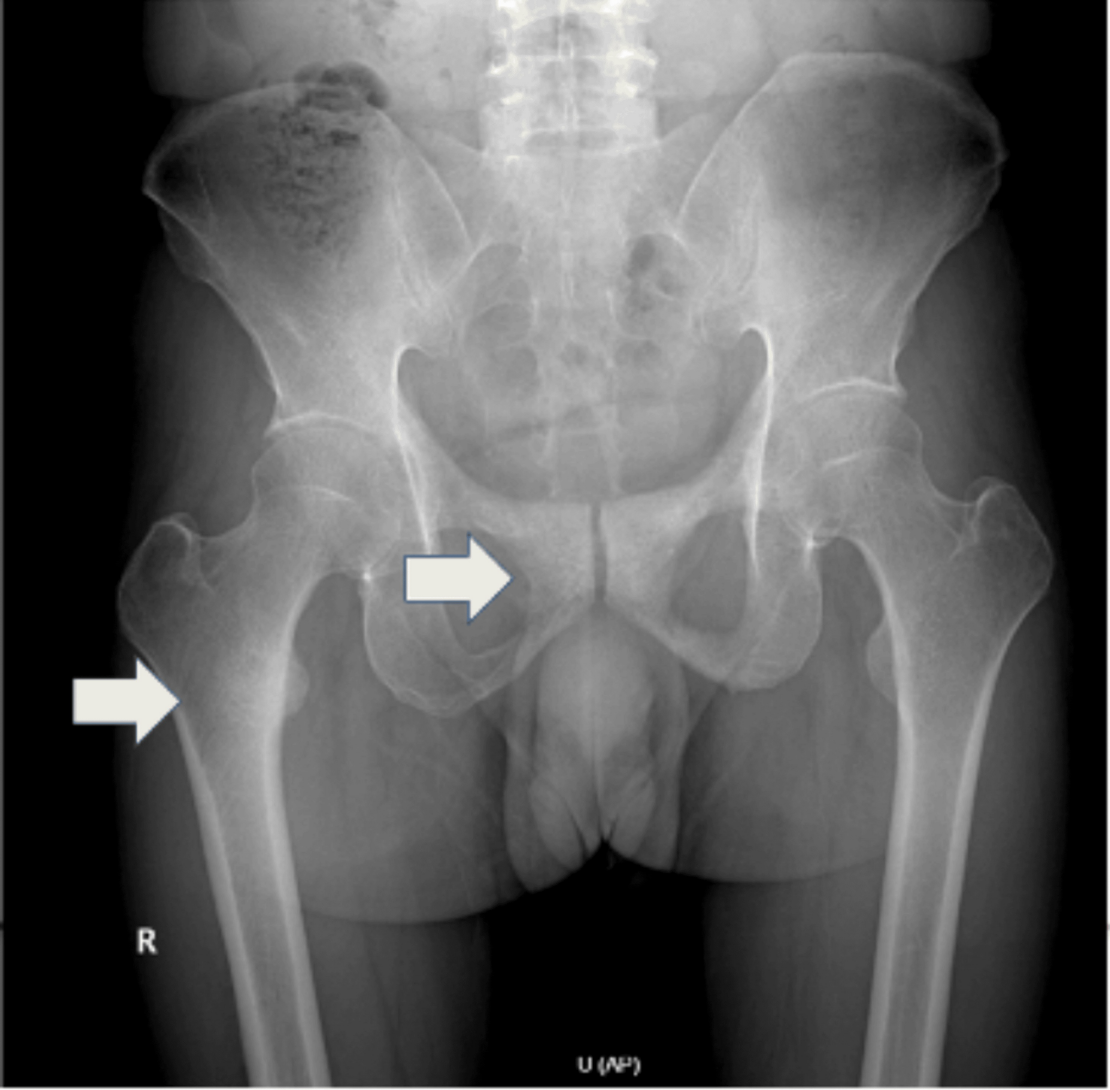
Plain radiograph of the pelvis and femur indicating metastatic lesions. White arrows show metastatic involvement of the right femoral lesser trochanter, bilateral ischium, and pubic rami.

**Figure 3:**
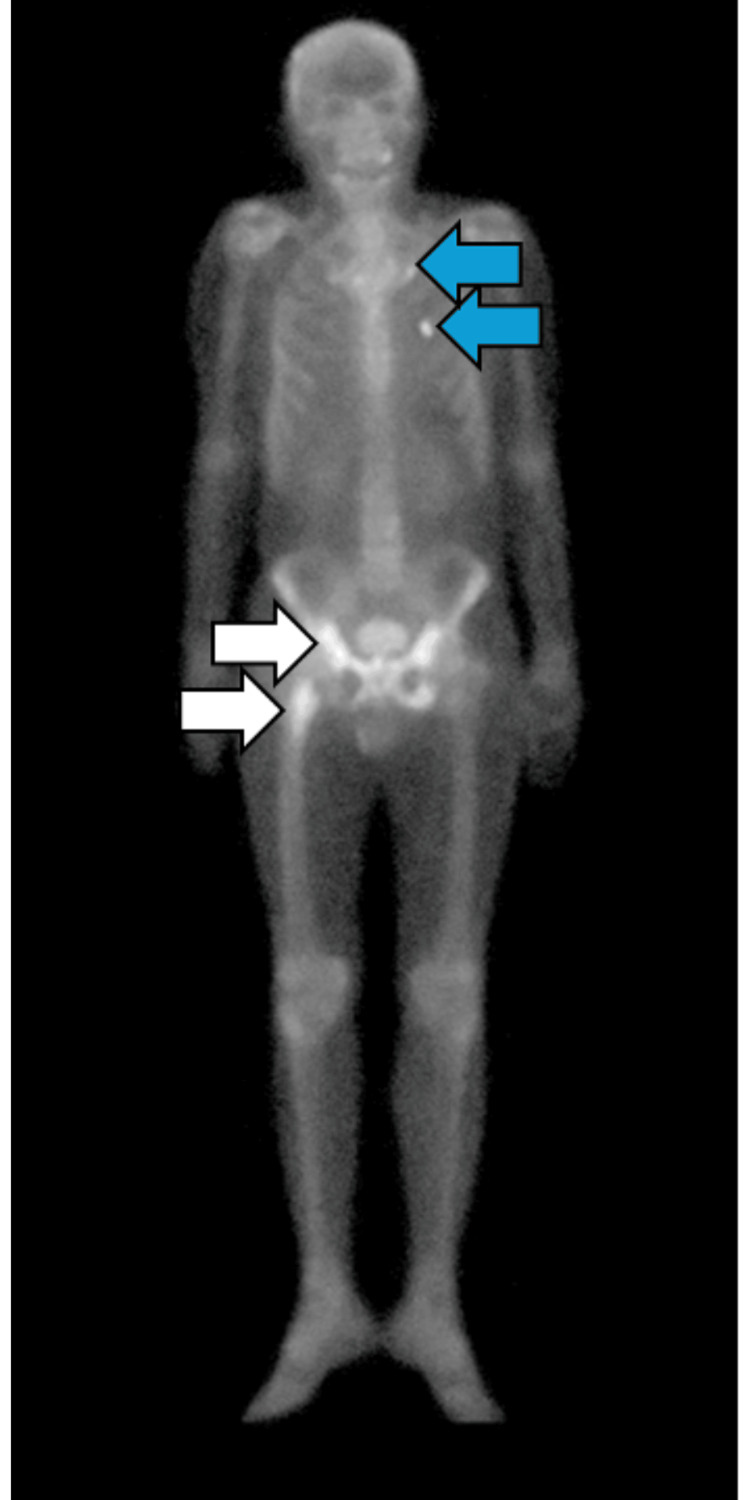
Bone scintigraphy demonstrating osseous metastases. White arrows show increased radiotracer uptake in the right pelvis and proximal right femur, while blue arrows show calcifications at the costochondral junctions of the left first and third ribs.

Further imaging with plain radiography demonstrated metastatic lesions involving the lesser trochanter of the right femur, as well as the right and left ischium and pubic rami. These radiographic findings are illustrated in Figure 2. A bone scan performed in the same month confirmed metastatic involvement of the right pelvis and femur, along with additional findings suggestive of calcification at the costochondral junctions of the first and third left ribs. The bone scintigraphy results are depicted in Figure 3.

**Figure 4:**
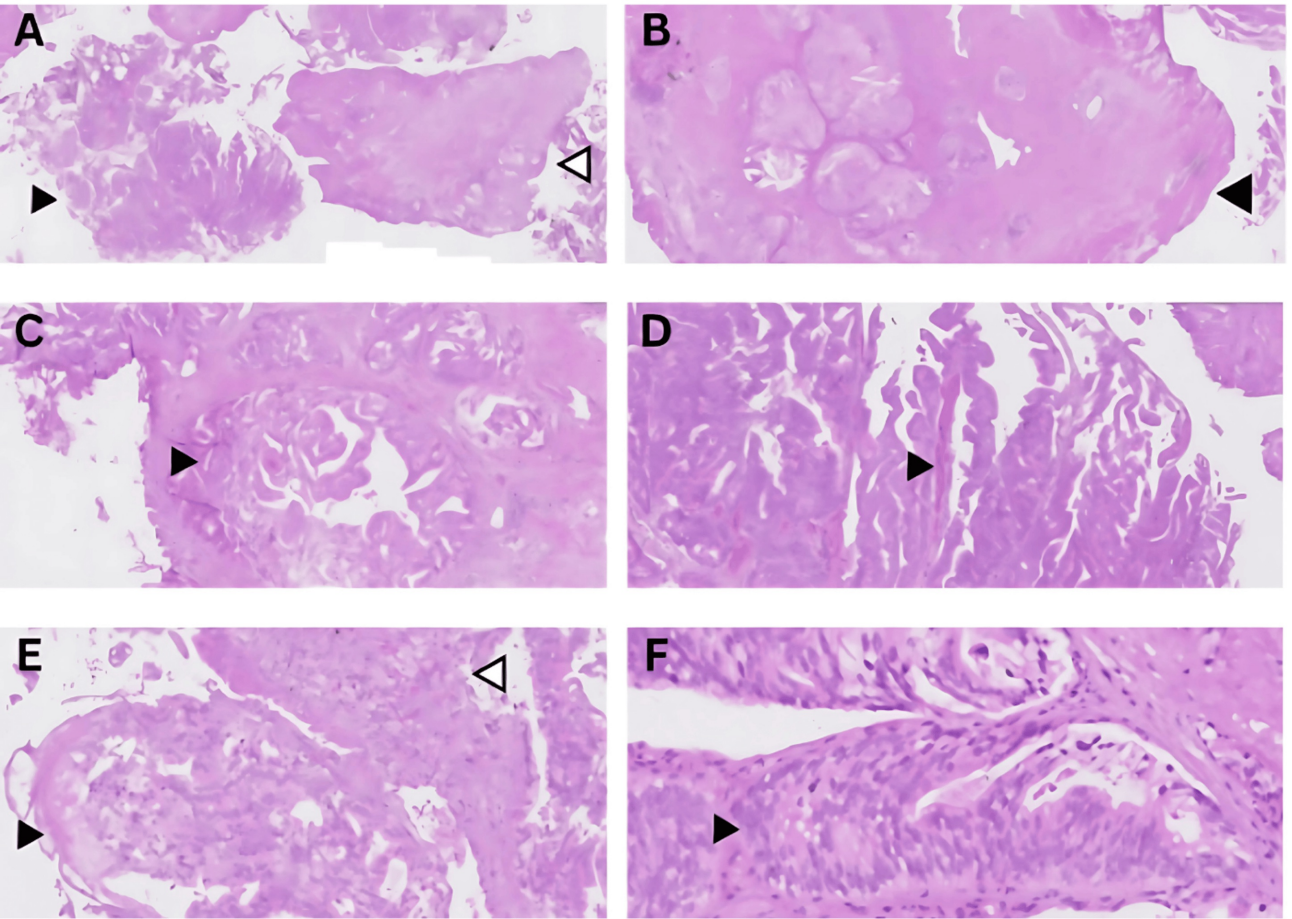
Histopathological examination of transurethral resection specimens involving the prostate and bladder neck. (A) Fragments of tissue consist of tumor tissue (black arrowhead) and fibromuscular tissue (white arrowhead); H&E 1x. (B) Tumor tissue infiltrating the bladder (note the muscle tissue at the edge shown by the black arrowhead); H&E 2x. (C) Tumors are arranged intraglandularly with slit-like openings (black arrowhead); H&E 5x. (D) papillary with true fibrovascular core (black arrowhead); H&E 5x. (E) Tumors are arranged in cribriform (black arrowhead) and solid (white arrowhead); H&E 10x. (F) The tumor is composed of tall columnar cells with pseudostratified nuclei (black arrowhead); the cytoplasm is amphophilic; H&E 20x. H&E: hematoxylin and eosin stain

Histopathological analysis of the TUR specimen showed well-differentiated adenocarcinoma. Microscopic examination revealed glandular structures consistent with ductal morphology. No evidence of necrosis was identified. The representative histological section is shown in Figure 4. The differential diagnoses considered were (1) prostate adenocarcinoma with bladder invasion, (2) primary bladder adenocarcinoma and (3) adenocarcinoma infiltrating from the lower gastrointestinal tract.

**Figure 5:**
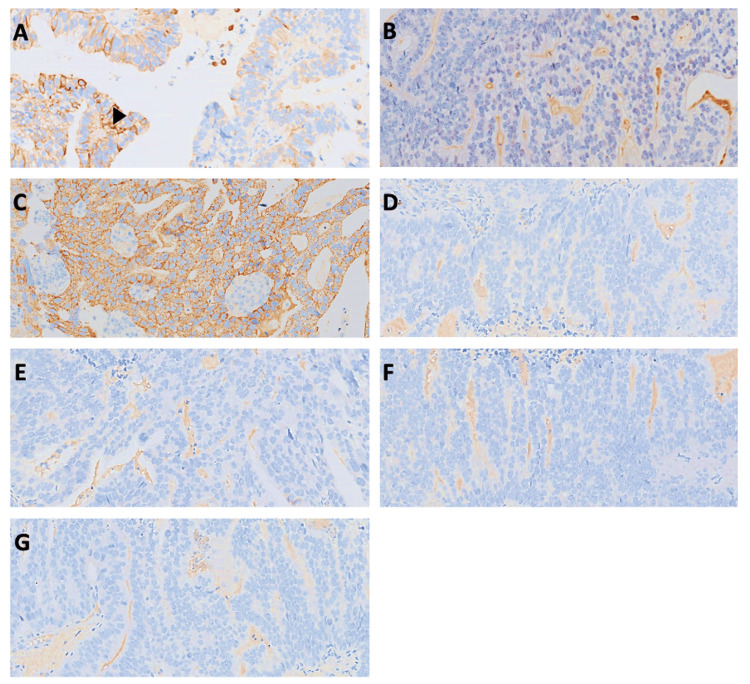
Immunohistochemistry profile of the tumor. (A) Patchy positive CK 7 (black arrowhead); 20x. (B) Negative CK20; 20x. (C) Diffuse positive AMACR, cytoplasmic staining; 20x. (D) Negative PSA; 20x. (E) Negative GATA 3; 20x. (F) Negative p63; 20x. (G) Negative CDX2; 20x. AMACR: α-Methylacyl-CoA racemase; CDX2: caudal-type homeobox transcription factor 2; CK7: cytokeratin 7; CK20: cytokeratin 20; GATA3: GATA-binding protein 3; p63: tumor protein 63; PSA: protein-specific antigen

To further clarify the origin of the tumor, immunohistochemical (IHC) staining was performed. The tumor cells showed patchy positivity for cytokeratin 7 (CK7), strong cytoplasmic staining for alpha-methyl acyl-CoA racemase (AMACR), and were negative for PSA, cytokeratin 20 (CK20), GATA-binding protein 3 (GATA3), tumor protein 63 (p63), and caudal-type homeobox transcription factor 2 (CDX2). The immunohistochemical staining patterns are demonstrated in Figure 5. Based on the morphological and immunophenotypic profile, the final diagnosis was confirmed as ductal adenocarcinoma of the prostate, Gleason score 4+4=8 (grade group 4), with histological evidence of bladder wall infiltration.

**Figure 6:**
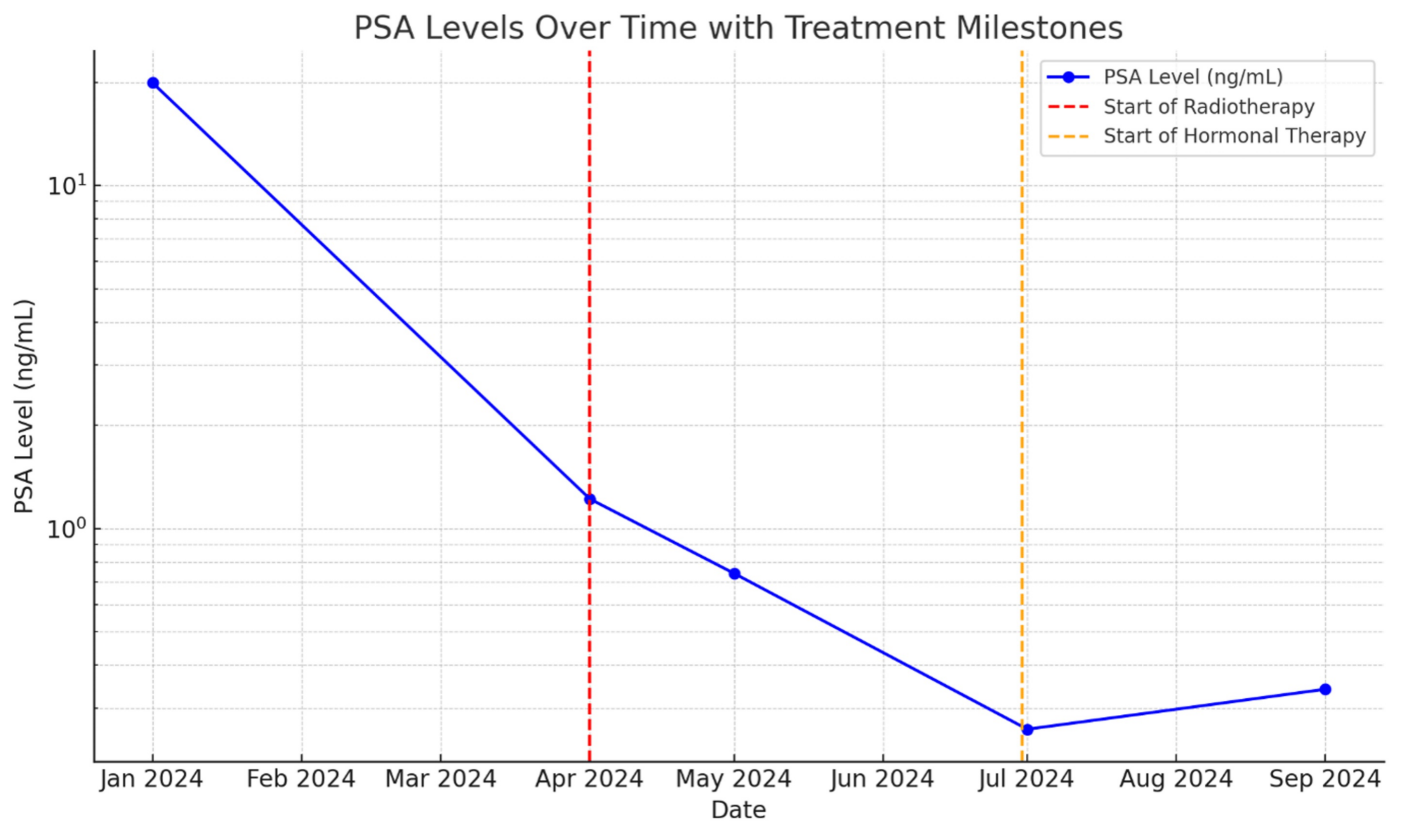
The line graph of PSA level in the case. PSA: prostate-specific antigen

The patient underwent a total dose of 74 Gy in 37 fractions of external beam radiotherapy using intensity-modulated radiation therapy (IMRT), consistent with standard dosing protocols for high-risk prostate cancer (typically totaling 70-78 Gy in conventional fractions). Following completion of radiotherapy, he was started on antiandrogen therapy with bicalutamide 150 mg daily, which was later substituted with monthly leuprolide acetate injections. For pain control, the patient received 10 mg of morphine twice daily. The line graph of PSA-level information can be seen in Figure 6. Serial follow-up demonstrated a consistent biochemical response. PSA levels declined from 19.94 ng/mL in January 2024 to 1.22 ng/mL in April 2024, 0.74 ng/mL in May 2024, 0.26 ng/mL in July 2024, and 0.34 ng/mL in September 2024. The line graph of PSA-level information can be seen in Figure 6.

## Discussion

Although PDA has been documented to exhibit both bladder invasion and skeletal metastases, the simultaneous occurrence of both is rarely reported. PDA is known for its locally advanced presentation (T3/T4 stages) and its capacity for distant metastasis at diagnosis. Visceral metastases, including involvement of the bladder, have been reported in 23.2% to 44.2% of metastatic PDA cases [7,8]. The current case reflects this aggressive biological behavior, with both bladder infiltration and femoral bone metastases present at initial staging, underscoring the tumor's potential for atypical and extensive metastatic spread [8,9].

The typical clinical presentation of PDA includes lower urinary tract symptoms such as obstructive voiding, hematuria, and dysuria, largely due to its periurethral and prostatic urethral origin [7,9]. This patient presented with urinary complaints and significant weight loss, both consistent with prior reports. Unlike acinar adenocarcinoma, PDA often presents at an advanced stage and may be associated with disproportionately low PSA levels [2,9]. However, in this case, the PSA was elevated (19.94 ng/mL), which is more consistent with high tumor burden, as no histopathological evidence of acinar adenocarcinoma was observed in the transurethral resection specimen. Therefore, the elevated PSA may reflect extensive tumor volume or sampling limitations that missed a potential minor acinar component. The clinical aggressiveness of PDA is well established, with a reported 4.6-fold increased risk of metastasis and a cancer-specific mortality rate similar to that of Gleason score 4+4 acinar adenocarcinoma [1,10]. The rapid progression and extent of disease in this patient are consistent with the known clinical trajectory of PDA [2,7,9]. 

Definitive diagnosis of PDA hinges on histopathological evaluation demonstrating characteristic ductal morphology, such as papillary or cribriform architectural patterns. Imaging modalities, particularly computed tomography (CT), magnetic resonance imaging (MRI), and bone scintigraphy, play an essential role in staging. In this case, abdominopelvic CT revealed contiguous bladder and prostate involvement, while a bone scan confirmed metastatic lesions. The diagnostic tissue was obtained via TUR, a common and effective method for obtaining representative samples when PDA is suspected [9,11,12].

Histologically, PDA is typified by tall pseudostratified columnar epithelial cells forming papillary and cribriform structures. This case showed glandular architecture in keeping with those features (Figure 4). Immunohistochemistry (IHC) plays a crucial role in distinguishing PDA from its histological mimics, particularly primary bladder adenocarcinoma or secondary invasion from gastrointestinal sources. Classical IHC findings in PDA include positivity for PSA and AMACR, with variable CK7 and CK20 expression. Interestingly, this case demonstrated patchy CK7 positivity, strong AMACR staining, and negative results for PSA, GATA3, p63, CK20, and CDX2 [11,12]. Although prostate adenocarcinoma, including its ductal subtype, is generally reported as CK7-negative, this case demonstrated positive CK7 expression. This finding may be attributed to urothelial differentiation, mixed histological features, or a rare phenotypic variant. Another possibility that must be considered is metastasis from a primary tumor at another CK7-positive site. Therefore, CK7 expression should be interpreted with caution and evaluated alongside a comprehensive immunohistochemical panel to ensure diagnostic precision [13,14]. 

Additionally, while PSA negativity is atypical for prostate adenocarcinoma, the absence of GATA3, a marker of urothelial origin, along with the positive AMACR, supported a diagnosis of prostatic ductal adenocarcinoma. In rare cases, urothelial carcinoma with glandular differentiation or metastatic colorectal adenocarcinoma may exhibit overlapping immunophenotypes; however, the absence of GATA3 and CDX2 in this case helped exclude those possibilities. This highlights the importance of combining morphology with a broad IHC panel to ensure accurate diagnosis in cases with atypical marker expression. [9,11,12]. 

Management of PDA typically involves a multimodal approach comprising surgery, radiation therapy, and androgen deprivation therapy (ADT). In this case, the patient received external beam radiotherapy followed by hormonal therapy, initially bicalutamide, later substituted with leuprolide acetate. A notable biochemical response was observed, with PSA levels declining from 19.94 ng/mL to 0.34 ng/mL over an eight-month period. This reinforces the potential benefit of multimodal treatment, although clinical and biochemical follow-up remains essential given PDA’s aggressive nature and recurrence risk. According to current guidelines, treatment of high-risk and locally advanced prostate cancer, including ductal variants, should incorporate external beam radiotherapy combined with long-term ADT. There is growing evidence supporting the use of novel androgen receptor-targeted therapies or chemotherapy in metastatic or hormone-refractory cases, although data specific to PDA remains limited due to its rarity [1]. 

While this response is encouraging, PDA is known for its variable sensitivity to therapy and its tendency for early recurrence and progression. The overall prognosis in metastatic PDA remains poor, with median survival often reported at less than three years [8,9]. Several factors may influence prognosis in these cases, including the extent and site of metastasis, baseline PSA level, response to initial therapy, and performance status of the patient. In this case, the early biochemical response is favorable, but continued monitoring is essential given the unpredictable clinical course of PDA [8,9]. 

## Conclusions

This case illustrates the aggressive clinical course and diagnostic complexity of ductal adenocarcinoma of the prostate with concurrent bladder invasion and bone metastases. Despite atypical immunohistochemical findings, particularly PSA negativity, the final diagnosis was supported by morphologic features and the exclusion of other primary sites through IHC. The patient’s initial therapeutic response highlights the potential efficacy of multimodal treatment, although long-term outcomes in such cases remain guarded. This report underscores the importance of considering PDA in patients presenting with lower urinary tract symptoms and unusual metastatic patterns and reinforces the diagnostic value of integrated histological and immunohistochemical analysis.
